# A retrospective propensity-score-matched cohort study of the impact of procalcitonin testing on antibiotic use in hospitalized patients during the first wave of COVID-19

**DOI:** 10.1093/jac/dkae246

**Published:** 2024-09-09

**Authors:** Jonathan A T Sandoe, Detelina Grozeva, Mahableshwar Albur, Stuart E Bond, Lucy Brookes-Howell, Paul Dark, Joanne Euden, Ryan Hamilton, Thomas P Hellyer, Josie Henley, Susan Hopkins, Philip Howard, Daniel Howdon, Chikezie Knox-Macaulay, Martin J Llewelyn, Wakunyambo Maboshe, Iain J McCullagh, Margaret Ogden, Helena K Parsons, David G Partridge, Neil Powell, Graham Prestwich, Dominick Shaw, Bethany Shinkins, Tamas Szakmany, Emma Thomas-Jones, Stacy Todd, Robert M West, Enitan D Carrol, Philip Pallmann

**Affiliations:** Department of Microbiology, The General Infirmary at Leeds, Leeds, UK; Healthcare Associated Infection Group, Leeds Institute of Medical Research, University of Leeds, Leeds, UK; Centre for Trials Research, College of Biomedical and Life Sciences, Cardiff University, Cardiff, UK; Department of Infection Sciences, Southmead Hospital, North Bristol NHS Trust, Bristol, UK; Pharmacy Department, Mid Yorkshire Teaching NHS Trust, Wakefield, UK; Centre for Trials Research, College of Biomedical and Life Sciences, Cardiff University, Cardiff, UK; Division of Immunology, Immunity to Infection and Respiratory Medicine, Faculty of Biology, Medicine and Health, The University of Manchester, Manchester, UK; Centre for Trials Research, College of Biomedical and Life Sciences, Cardiff University, Cardiff, UK; Antibiotic Research UK, York, UK; School of Pharmacy, De Montfort University, Leicester, UK; Royal Victoria Infirmary, Newcastle and Freeman Hospital, Newcastle-upon-Tyne Hospital NHS Foundation Trust, Newcastle-upon-Tyne, UK; Translational and Clinical Research Institute, Faculty of Medical Sciences, Newcastle University, Newcastle, UK; School of Social Sciences, Cardiff University, Cardiff, UK; UK Health Security Agency, London, UK; NHS England and NHS Improvement, North-East and Yorkshire Region, UK; Leeds Institute for Health Sciences, University of Leeds, Leeds, UK; Public and Patient Involvement Representative, Centre for Trials Research, Cardiff University, Cardiff, UK; Brighton and Sussex Medical School, University of Sussex and University Hospitals Sussex NHS Foundation Trust, Brighton, UK; Centre for Trials Research, College of Biomedical and Life Sciences, Cardiff University, Cardiff, UK; Royal Victoria Infirmary, Newcastle and Freeman Hospital, Newcastle-upon-Tyne Hospital NHS Foundation Trust, Newcastle-upon-Tyne, UK; Translational and Clinical Research Institute, Faculty of Medical Sciences, Newcastle University, Newcastle, UK; Public and Patient Involvement Representative, Centre for Trials Research, Cardiff University, Cardiff, UK; Department of Microbiology, Laboratory Medicine, Northern General Hospital, Sheffield Teaching Hospitals NHS Foundation Trust, Sheffield, UK; Department of Microbiology, Laboratory Medicine, Northern General Hospital, Sheffield Teaching Hospitals NHS Foundation Trust, Sheffield, UK; Pharmacy Department, Royal Cornwall Hospitals NHS Foundation Trust, Truro, UK; Public and Patient Involvement Representative, Centre for Trials Research, Cardiff University, Cardiff, UK; Leicester NIHR Biomedical Research Centre and Department of Respiratory Sciences, University of Leicester, Leicester, UK; Leeds Institute for Health Sciences, University of Leeds, Leeds, UK; Division of Health Sciences, Warwick Medical School, University of Warwick, Coventry, UK; Critical Care Directorate, Aneurin Bevan University Health Board, Cwmbran, UK; Department of Anaesthesia, Intensive Care and Pain Medicine, Division of Population Medicine, Cardiff University, Cardiff, UK; Centre for Trials Research, College of Biomedical and Life Sciences, Cardiff University, Cardiff, UK; Tropical and Infectious Disease Unit, Liverpool University Hospitals NHS Foundation Trust, Liverpool, UK; Leeds Institute for Health Sciences, University of Leeds, Leeds, UK; Department of Clinical Infection, Microbiology and Immunology, Institute of Infection, Veterinary and Ecological Sciences, University of Liverpool, Liverpool, UK; Centre for Trials Research, College of Biomedical and Life Sciences, Cardiff University, Cardiff, UK

## Abstract

**Background:**

Procalcitonin (PCT) is a blood marker used to help diagnose bacterial infections and guide antibiotic treatment. PCT testing was widely used/adopted during the COVID-19 pandemic in the UK.

**Objectives:**

Primary: to measure the difference in length of early (during first 7 days) antibiotic prescribing between patients with COVID-19 who did/did not have baseline PCT testing during the first wave of the pandemic. Secondary: to measure differences in length of hospital/ICU stay, mortality, total days of antibiotic prescribing and resistant bacterial infections between these groups.

**Methods:**

Multi-centre, retrospective, observational, cohort study using patient-level clinical data from acute hospital Trusts/Health Boards in England/Wales. Inclusion: patients ≥16 years, admitted to participating Trusts/Health Boards and with a confirmed positive COVID-19 test between 1 February 2020 and 30 June 2020.

**Results:**

Data from 5960 patients were analysed: 1548 (26.0%) had a baseline PCT test and 4412 (74.0%) did not. Using propensity-score matching, baseline PCT testing was associated with an average reduction in early antibiotic prescribing of 0.43 days [95% confidence interval (CI): 0.22–0.64 days, *P* < 0.001) and of 0.72 days (95% CI: 0.06–1.38 days, *P* = 0.03] in total antibiotic prescribing. Baseline PCT testing was not associated with increased mortality or hospital/ICU length of stay or with the rate of antimicrobial-resistant secondary bacterial infections.

**Conclusions:**

Baseline PCT testing appears to have been an effective antimicrobial stewardship tool early in the pandemic: it reduced antibiotic prescribing without evidence of harm. Our study highlights the need for embedded, rapid evaluations of infection diagnostics in the National Health Service so that even in challenging circumstances, introduction into clinical practice is supported by evidence for clinical utility.

**Study registration number:**

ISRCTN66682918.

## Introduction

Even before the coronavirus disease 2019 (COVID-19) pandemic, community acquired pneumonia (CAP) was a prevalent and serious disease, with evidence that early administration of antibiotics improves outcomes. It remains challenging to differentiate bacterial from viral causes of lower respiratory tract infection (LRTI) on clinical grounds and antibiotics are often administered inappropriately. A lack of reliable, rapid diagnostics to determine the pathogen in patients with LRTI has led to measurement of the inflammatory marker procalcitonin (PCT) in blood samples to help diagnose bacterial infections and aid antibiotic prescribing decisions, particularly in respiratory tract infections.^1^ Two systematic reviews of the diagnostic accuracy of PCT for differentiating bacterial from viral infection have found similar specificity (73% and 76%) but very different sensitivity (92% and 55%).^2,3^ In spite of these contradictory findings, a further systematic review of PCT-guided treatment in acute respiratory infections (undertaken pre-COVID-19) found that 30-day mortality was significantly lower in patients with PCT-guided treatment than in control patients.^4^ PCT use was also associated with a significant 2.4-day average reduction in antibiotic exposure and a reduction in antibiotic-related side-effects.

During the first wave of the COVID-19 pandemic, there was concern about potential overuse of antibiotics and a negative impact on antimicrobial resistance. International guidelines included recommendations for empirical antibiotic therapy for patients with suspected or confirmed severe COVID-19, COVID-19-related sepsis and CAP^5^ and a high proportion of patients with COVID-19 (75%) were prescribed antibiotics.^6^ In an attempt to strengthen antimicrobial stewardship in times of uncertainty, many National Health Service (NHS) hospitals in the UK used PCT testing to assist antibiotic prescribing in patients with COVID-19,^7^ and some centres reported the value of PCT in reducing antibiotic use.^8^ Local guidance decisions to use PCT were contrary to National Institute for Health and Care (NICE) recommendations, which acknowledged the lack of evidence and promoted engagement with research.^9^ Large, publicly funded, randomized controlled trials^10–12^ are currently underway in the UK to assess the impact of PCT testing on antibiotic use but these are not specifically focused on patients with COVID-19. We therefore conducted a multi-centre observational assessment of the impact of PCT testing on antibiotic use and patient outcomes in patients with COVID-19.

## Objectives

Our primary objective was to measure the difference in days on early antibiotic treatment between patients with COVID-19 who did/did not have baseline PCT testing. Secondary objectives were to measure differences in length of hospital/ICU stay, mortality, total days of antibiotic treatment and resistant bacterial infections between these two groups.

## Methods

### Ethics and approvals

The study was approved by the NHS Research Ethics Committee (West Midlands—Solihull Research Ethics Committee, reference 21/WM/0052), Health Research Authority and Health and Care Research Wales on 3 March 2021. Study registration: ISRCTN66682918. The Procalcitonin: Evaluation of Antibiotic prescribing in COVID-19 Hospitalized patients (PEACH) study collected patient data in accordance with the notice under Regulation 3(4) of the Health Service (Control of Patient Information) Regulations 2002 (COPI).

### Study design

This was a multi-centre, retrospective, observational, cohort study using patient-level clinical data and designed/reported with consideration of STROBE criteria.^13^ A study protocol and statistical analysis plan have been previously published.^14^

### Setting

Eleven NHS acute hospital Trusts/Health Boards in England and Wales took part in the study including both teaching and district general hospitals. Rates of PCT use in patients with COVID-19 during the study period varied considerably between these sites.^7^ Patients were followed up until discharge/death. The study period was between 1 February and 30 June 2020.

### Participants

Potentially eligible patients were identified from institutional databases/medical records by the clinical teams at each participating organization. Inclusion criteria were that patients ≥16 years had been admitted to a participating organization and had a confirmed positive PCR COVID-19 test (according to local testing procedures) during the study period. Exclusion criteria were second and subsequent admissions after index admission with COVID-19.

### Variables

The collected variables have been published previously.^14^ Sex assigned at birth was recorded as documented in medical records. Day 1 of COVID-19 infection was considered the day of the first positive sample. ‘Baseline’ variables were those collected on/around the time of COVID-19 diagnosis, i.e. day 1 (±1 day). The primary outcome was days of early antibiotics therapy (≤day 7 after first positive COVID-19 test sample during the study period). Early ICU admission was defined as admitted to/already on ICU at baseline. Secondary outcomes were: total days of antibiotic treatment; days of late (>day 7 after positive COVID-19 test sample) antibiotic treatment; 30-day mortality; 60-day mortality; length of stay (hospital and ICU after day 3) and antimicrobial-resistant secondary bacterial infection (onset after day 3). Antimicrobial resistance was defined as resistant to ≥3 antibiotic classes (with sensitivity analyses for resistance to ≥1 or ≥2 classes). Descriptive outcomes were frequency of PCT testing and types of secondary bacterial infection.

### Data sources

Routine clinical data from a patient’s episode of hospitalization were collected manually from individual patient medical records/NHS databases. Data were de-identified at source and entered into a bespoke study database developed by the Centre for Trials Research (CTR) (MACRO version 4.9.1) and hosted on Cardiff University secure servers. Some study variables were preferentially collected directly from primary care medical records and, if not available, from their secondary care records.

### Data quality control

Extensive data quality control was performed. If paired start/end dates were incomplete, the pair was excluded from the analysis (e.g. hospital admission date missing while discharge date was present). If logical inconsistencies were observed, such data points were excluded (e.g. antibiotics start date after end date, ICU discharge date after hospital discharge date). Self-evident corrections were made where appropriate in cases of obvious typographical errors (e.g. year 2020 entered as 2002). If blood laboratory tests were performed repeatedly on the same date for the same patient, the highest value was used.

### Statistical analysis

#### Sample size

Based on a minimal difference in antibiotic duration of 1 day^10,11,15^ between baseline PCT-tested and non-PCT-tested patients, and an assumed standard deviation (SD) of 6 days, 1500 matched patients were estimated to provide 90% power when using a two-sample t-test with two-sided 5% type I error rate. To account for the reduction in effective sample size due to propensity-score matching, and expecting fewer patients having PCT than not, we aimed to source data for ∼7000 patients.

#### Bias

To reduce the risk of bias, consecutive patients fulfilling the eligibility criteria were included. Identification of subjects was carried out without prior knowledge of outcomes or PCT testing status and by separate teams from those carrying out the analysis. Potential confounding factors for inclusion in the propensity-score analysis, i.e. those potentially influencing both the outcomes and the decision to use PCT testing, were agreed in advance of analysis and published.^14^ Objective criteria for study variables were agreed in advance.

#### Descriptive statistics

Descriptive (e.g. means or medians with SDs for continuous variables, frequencies and percentages for binary or categorical variables) and graphical summaries (e.g. histograms) were generated according to baseline PCT testing (yes or no).

#### Propensity-score matching

To assess the effect of PCT testing on antibiotic prescribing and patient outcomes while ensuring an even distribution of important confounders between groups, propensity-score matching was used. Patients who did/did not receive PCT testing at baseline were matched. Propensity-score matching was used to reduce potential differences between the ‘tested’ (i.e. PCT test at baseline) and ‘untested’ patients (i.e. no PCT test at baseline) on characteristics deemed prognostic of clinical endpoints.^S1^

The following covariates were used in the propensity-score modelling: age, sex, number of comorbidities, smoking status, ethnicity, index of multiple deprivation, quick Sequential (Sepsis-Related) Organ Failure Assessment (qSOFA) score^S1^; Confusion, Uraemia, Respiratory rate, Blood pressure, age ≥65 (CURB-65) score^S2^ for pneumonia severity; National Early Warning Score 2 (NEWS2)^S3^; 4C Mortality score^S4^; presence of early secondary bacterial infection; admission to ICU at baseline; blood laboratory tests (C-reactive protein, white blood cell count, neutrophil count, D-dimer, troponin) at baseline and chest radiograph imaging [uncoded, definite COVID-19, normal, indeterminate, non-COVID-19 chest radiograph abnormalities (lobar pneumonia, pleural effusions, pulmonary oedema or other patterns)] at baseline (i.e. performed ± day of a positive COVID-19 test), as well as indicator variables denoting missing blood test data.

Propensity-score-matched data were generated with the aim of estimating the average effect of baseline PCT testing on a population of patients similar to those who received PCT testing at baseline, but who did not, thus measuring the effect withdrawal of baseline PCT testing might have; this average effect of testing on the tested (ATT, propensity-score matching to estimate the average effect of baseline PCT testing on the tested population) was considered the estimand for the primary analysis. In a secondary analysis we estimated the average effect of baseline PCT testing on a population of patients like those who did not receive PCT testing at baseline, thus measuring the effect of introduction of baseline PCT testing to those who did not have it (i.e. the average effect of testing on the untested, ATU). Different methods for generating the propensity scores were explored, balance diagnostics were used to check the adequacy of matching and the optimal method selected. Checking adequacy of matching involved examining if the distributions of measured baseline covariates were similar between patients who received the PCT test at baseline and those who did not, based on the standardized mean difference and Kolmogorov–Smirnov statistic with 0.1 used as a threshold for acceptable covariate balance.^S5^ Plots of standardized mean difference and Kolmogorov–Smirnov statistics for all covariates for both ATT and ATU are presented in Figures S1 and S2 (available as Supplementary data at *JAC* Online).

For the ATT estimand, optimal full matching was used, with the propensity score estimated with probit regression. For the ATU estimand, nearest neighbour matching without replacement was used, with the propensity score estimated with probit regression and a calliper (i.e. the used difference in the propensity scores between matched subjects) fixed at 0.02. G-computation in the matched sample was used to estimate the ATT and ATU. A cluster-robust variance was used to estimate its standard error with matching stratum membership as the clustering variable.

#### Main analysis

Regression modelling was used to examine whether baseline PCT testing affected the days on early antibiotic treatment and other outcomes. Models had treatment (i.e. PCT test done at baseline or not) as predictor variable and also included the matching weights derived from the propensity scores. Results are presented as effect estimates with 95% confidence intervals (CIs) and *P* values for both the average effect on the tested (ATT, primary analysis) and the untested population (ATU, secondary analysis).^S5^ The type of analysis model for the propensity-score-matched data depended on the type of outcome i.e. logistic regression for binary outcomes (ICU admission, mortality, SBI) and linear regression for continuous outcomes (days on early/late/total antibiotics, length of hospital stay). The risk of unmeasured confounding was quantified using E-values.^S6^ Subgroup analyses included testing if the number of performed PCT tests or being in ICU at baseline were associated with the duration of early antimicrobial treatment.

#### Statistical software

STATA version 17 was used for data management and descriptive statistics. Propensity-score analyses were performed in R version 4.3.1,^S7^ with add-on packages ‘MatchIt’ version 4.5.4^S8^ for propensity-score matching, ‘cobalt’^S9^ for assessing covariate imbalance, ‘marginaleffects’^S10^ to perform g-computation and ‘ggplot2’ ^S11^ for graphical presentation.

## Results

### Propensity-score matching

Nearest neighbour propensity-score matching with and without replacement and optimal full matching with different parameters were tested and found to be successful for primary (ATT) analysis while optimal full matching was not successful for secondary (ATU) analysis (Figures S1 and S2).

### Participants

**Figure 1.**
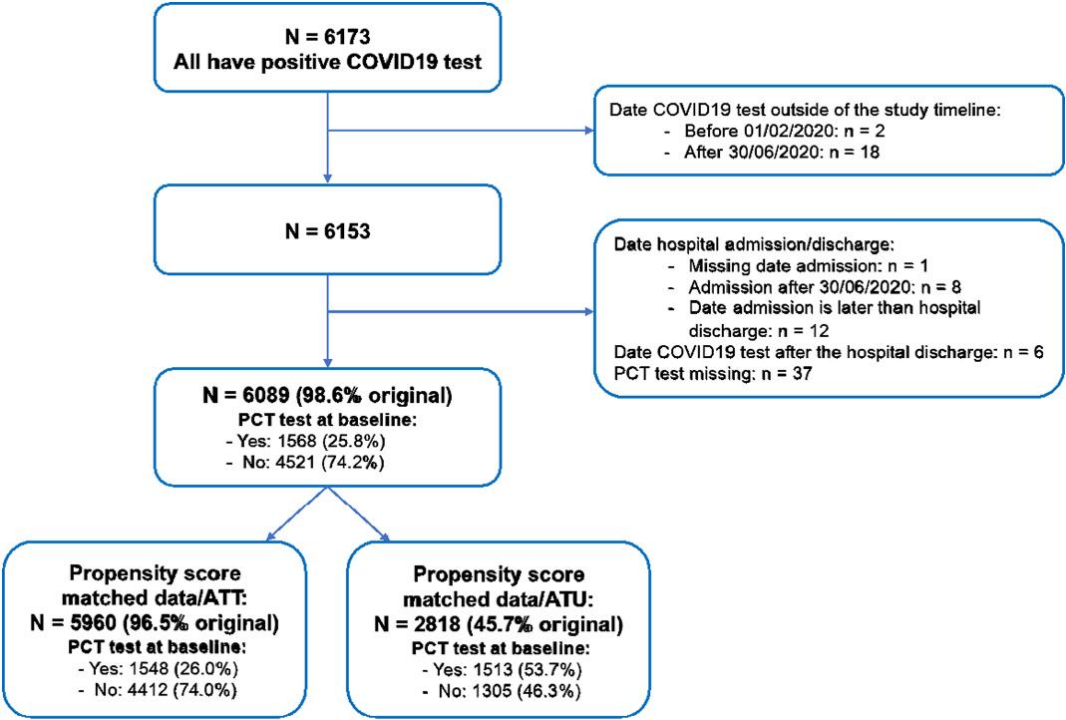
Recruitment flowchart and description of reasons for exclusions: (a) proportion of second-line therapies and (b) success rate of second-line therapies. This figure appears in colour in the online version of *JAC* and in black and white in the print version of *JAC*.

Data for 6173 individuals with a positive COVID-19 test were collected. After quality control, 6089 remained (patient characteristics in Tables S1 and S2). Data from 5960 of 6089 (97.9%) people were used for the propensity-score-matched primary (ATT analysis (Figure 1). The 5960 comprised 1548 (26.0%) participants with and 4412 (74.0%) without a PCT test at baseline (Table 1). 2818 people were included in the secondary (ATU) analysis, comprising 1513 (53.7%) with and 1305 (46.3%) without a PCT test at baseline.

**Table 1.** Distribution and frequency of procalcitonin (PCT) tests in the population used for primary propensity scoring (ATT)

	Frequency (%)
Baseline PCT	
Yes	1548 (26.0)
No	4412 (74.0)
PCT done at any time	
Yes	2200 (36.9)
No	3760 (63.1)
Number of PCT tests per person	
0	3760 (63.1)
1	1404 (23.6)
2 or more	796 (13.4)
Total	**5960**

### Descriptive analysis

Recruitment figures across participating organizations are in Table S3. Four organizations contributed with more than 13% each to the overall sample. A breakdown of the primary data sources is presented in Table S4. Most COVID-19 episodes were with community onset (72.9%), see Table S5. Descriptive statistics for the main participant characteristics are presented in Table 2 for the primary (ATT) analysis, and for the ATU analysis in Table S6. The number, frequency and breakdown of comorbidities per person is presented in Tables S7 and S8.

**Table 2.** Patient characteristics according to whether a PCT test was done at baseline or not, showing both propensity-score matched (ATT) and unmatched data

	PCT at baseline, unmatched sample (total *n* = 5960)		PCT at baseline, matched sample (total *n* = 5960)	
	No	Yes	No	Yes
	*n* (%)	*n* (%)	*n* (%)	*n* (%)
Age category				
16–49	472 (10.7)	188 (12.1)	512.3 (11.6)	188 (12.1)
50–59	445 (10.1)	233 (15.1)	649.8 (14.7)	233 (15.1)
60–69	617 (14.0)	251 (16.2)	813.9 (18.5)	251 (16.2)
70–79	1055 (23.9)	341 (22.0)	926.6 (21.0)	341 (22.0)
>80	1823 (41.3)	535 (34.6)	1509.4 (34.2)	535 (34.6)
Sex				
female	1970 (44.6)	674 (43.5)	1824.1 (41.3)	674 (43.5)
male	2434 (55.2)	872 (56.3)	2581.4 (58.5)	872 (56.3)
unknown	8 (0.2)	2 (0.1)	6.5 (0.2)	2 (0.1)
Ethnicity				
white	3380 (76.6)	1227 (79.3)	3557.5 (80.6)	1227 (79.3)
mixed	35 (0.8)	11 (0.7)	43.9 (1.0)	11 (0.7)
Asian	136 (3.1)	105 (6.8)	243.0 (5.5)	105 (6.8)
black	86 (2.0)	64 (4.1)	162.8 (3.7)	64 (4.1)
other	189 (4.3)	52 (3.4)	153.8 (3.5)	52 (3.4)
Unknown	586 (13.3)	89 (5.8)	251.1 (5.7)	89 (5.8)
Smoking status				
no	1864 (42.2)	619 (40.0)	1737.3 (39.4)	619 (40.0)
yes	206 (4.7)	64 (4.1)	151.1 (3.4)	64 (4.1)
ex-smoker	1208 (27.4)	351 (22.7)	984.3 (22.3)	351 (22.7)
Unknown	1134 (25.7)	514 (33.2)	1539.3 (34.9)	514 (33.2)
ICU admission at baseline				
no	4193 (95)	1333 (86.1)	3796.2 (86.0)	1333 (86.1)
yes	184 (4.2)	200 (12.9)	571.9 (13.0)	200 (12.9)
Unknown	35 (0.8)	15 (1.0)	43.9 (1.0)	15 (1.0)
Has the patient died (as of when the data were collected and input in the study database)?				
no	2374 (53.8)	921 (59.5)	2419.4 (54.8)	921 (59.5)
yes	2016 (45.7)	621 (40.1)	1965.4 (44.6)	621 (40.1)
unknown	22 (0.5)	6 (0.4)	27.1 (0.6)	6 (0.4)

### Primary outcome

**Figure 2.**
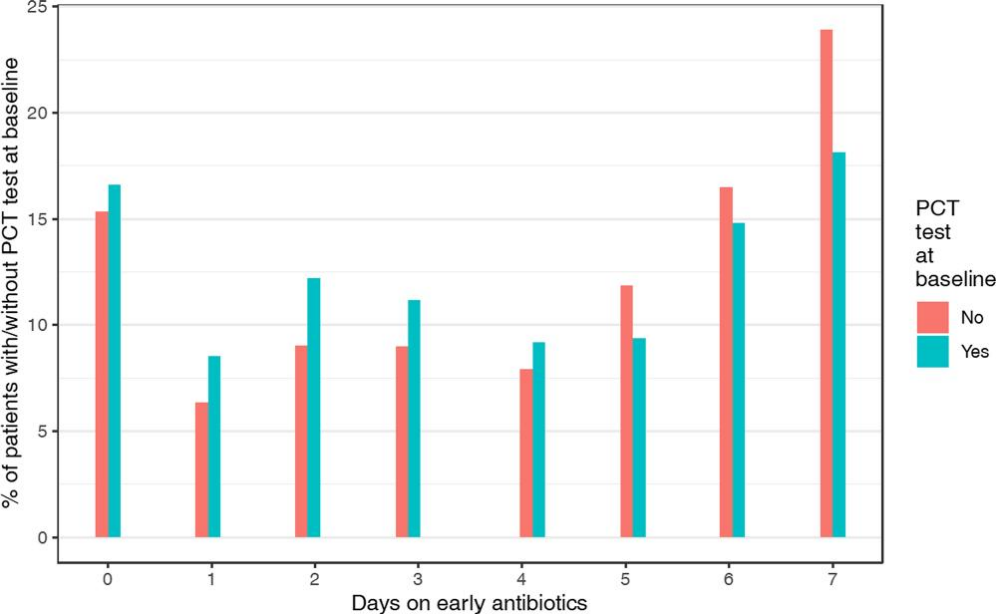
Histogram of days of primary outcome (early antibiotics, within first 7 days) according to whether a PCT test was done at baseline or not. Using propensity-score matching based on the ATT. This figure appears in colour in the online version of *JAC* and in black and white in the print version of *JAC*.

The mean number of days of early antibiotics in the matched data was 3.96 (SD 2.53). The spread of the number of days of early antibiotic therapy in the primary (ATT) analysis, broken down by PCT test status at baseline, is presented in Figure 2. The secondary (ATU) analysis is shown in Figure S3. The primary (ATT) analysis estimated a decrease in the average duration of early antibiotic therapy of 0.43 days (SE = 0.11, 95% CI 0.22–0.64, *P* < 0.001), per patient who had PCT testing at baseline compared to a (hypothetical) scenario in which they did not. A similar significant decrease of 0.30 days (SE = 0.10, 95% CI 0.11–0.49, *P* = 0.002) was estimated in the secondary (ATU) analysis.

### Secondary outcomes

#### Antibiotic prescribing

The estimated average effect of PCT testing at baseline on total antibiotic prescribing was a decrease of 0.72 days (SE = 0.33, 95% CI 0.06–1.38, *P* = 0.03), indicating the average effect of testing was to decrease the duration of total antibiotics by 0.72 days per tested patient compared to a (hypothetical) scenario with no testing. The spread of total days antibiotic therapy in the primary (ATT) analysis is presented in Figures S4 and S5. The secondary (ATU analysis did not show a statistically significant reduction in total duration. There was no effect of a baseline PCT on late antibiotic prescribing (decrease of 0.28 days, SE = 0.29, 95% CI 0.85- −0.27, *P* = 0.31) in the ATT analysis. While a PCT test at baseline was associated with reduced antibiotic duration, a single PCT test any time or multiple testing during the inpatient stay was associated with significant increases in both late and total antibiotic prescribing (Tables S9 and S10). With respect to late antibiotic prescribing in the secondary (ATU) analysis, similar to the primary (ATT) analysis, non-statistically significant results were observed.

#### Mortality and hospital/ICU length of stay (LOS)

For mortality at 30 and 60 days, and LOS (both in hospital and ICU) there were no statistically significant differences for patients who had baseline PCT testing or not in both matched analyses (Tables S11–S18 and Figures S6 and S7).

#### Resistant secondary bacterial infection

There were no statistically significant differences in the rate of antimicrobial-resistant secondary bacterial infections for patients who had baseline PCT testing or not in both matched analyses (Tables S19-S23).

#### Sensitivity analysis for unmeasured confounding

E-values were calculated to evaluate the robustness of our findings regarding potential unmeasured confounders.^S6^ The observed E-values for the primary ATT and secondary ATU analyses were 1.59 and 1.47, respectively.

#### Subgroup analysis

Subgroup analysis of patients admitted early to ICU, found that they were more likely to receive early antibiotic therapy and a baseline PCT was not associated with significant changes to antibiotic prescribing (Tables S24 and S25). This analysis is based on 384 patients who were admitted to ICU and 5526 who were not and as such is underpowered (Table S24).

## Discussion

Baseline PCT testing during COVID-19 was associated with a statistically significant reduction in antibiotic prescribing in the first seven days and in total days of antibiotic prescribing. There was no significant effect of baseline PCT testing on late antibiotic prescribing. There was no evidence of harm in terms of a significant effect of a baseline PCT on mortality at 30 and 60 days, hospital/ICU LOS or resistant secondary bacterial infection.

PCT was widely adopted in England and Wales during the first wave of the pandemic,^7^ contrary to subsequent NICE guidelines. We found that PCT measurement at baseline in hospitalized patients with COVID-19 was associated with fewer days of antibiotic prescribing, which is consistent with a systematic review examining the impact of PCT on antibiotic prescribing in respiratory tract infections before COVID-19 and with local hospital reports about PCT use during the pandemic.^8,16–18^ Confounding by severity of infection is a concern in many of the local studies, as well as their small sample size, both of which we attempted to address with the current design. The findings are also consistent with an analysis of aggregated data from English hospitals that found an initial fall in antibiotic use during the first wave of the pandemic in hospitals that used or introduced PCT, an effect that was slowly eroded over time.^19^

If PCT was responsible for the observed reduction in antibiotic use, its effect was small, on a patient level. This result is different from a meta-analysis of CAP cases which found a 2.45 day reduction in antibiotic duration in PCT-tested cases.^4^ This could be explained by the inconsistent way in which PCT was introduced with no or variable guidance on its use,^7^ or it may have little effect in this setting, similar to findings of Huang *et al*.^20^ It is possible that the effect of PCT-guided antibiotic use is dependent on the effectiveness of its implementation or that it may have greatest value in patients with low disease severity, where it gives clinicians more confidence to stop or withhold antibiotics. This idea is supported by the finding that a baseline PCT was not associated with antibiotic reductions in those admitted to ICU early in their admission, however, this analysis was underpowered due to the small proportion of patients admitted to ICU early in their hospital stay. Although the per-patient reduction in antibiotic duration associated with baseline PCT was small, the cumulative effect of these over the study period may have an important effect reducing selection for resistant bacteria, but this would need further study.

The perceived value of PCT is its ability to differentiate bacterial from viral infections. However, contrary to this, a range of peak PCT values have been demonstrated in patients with viral pneumonia indicating that viral infection can also increase PCT.^21^ PCT did not perform well to distinguish pure viral pneumonia from bacterial co-infection, but performed better as a prognostic rather than diagnostic marker.^21^ A recent analysis also found that PCT had poor diagnostic accuracy for detecting microbiologically confirmed bacterial infection at the time of presentation with COVID-19.^22^ In a mouse model of pure influenza A infection, PCT transcription in the lung was elevated and associated with increased expression of IL-6.^21^ When a human lung epithelial cell line was infected with influenza A, PCT expression correlated positively with viral load, IL-6 and IFN-γ.^21^ Thus, PCT expression is driven by the innate immune system; by bacterial antigens but also by tissue damage. The idea that higher PCT values relate to more tissue damage than just pathogen, is supported by the association of high levels with clinical deterioration in,^23^ and severity of, COVID-19.^24,25^ This means that PCT testing may help reduce antibiotics in the less severely unwell but might drive increased antibiotic use in the more severely unwell where diagnostic accuracy for bacterial infection appears to be worse. Although PCT did not appear to be harmful in terms of the objective measures of mortality and LOS, the lack of a positive impact on mortality contradicts data from before COVID-19, in a meta-analysis of data from 6708 patients.^4^

### Strengths and limitations

We obtained a large sample of patient data from geographically spread hospitals and various hospital types to improve generalizability. Our sampling strategy was purposeful, deliberately including institutions that did/did not introduce/use PCT testing during the first wave of the pandemic. This was a retrospective, observational, hospital record-based study with the associated problems of missing data, incorrectly recorded information, lack of randomization and reliance on data that were entered into the clinical records for the patients rather than purposefully collected and curated data. Although we took account of known confounding factors, we could not account for unknown confounders. The moderate E-value (1.59) suggests the primary analysis result is potentially sensitive to unmeasured confounders; the possibility of residual confounding cannot be excluded despite careful adjustment for available covariates during propensity-score matching. We aimed to reduce the risk of selection bias by including consecutive patients fulfilling the eligibility criteria. Data collecting/entering was separate to analysis. Collection of microbiology results was restricted to blood and respiratory samples; we may therefore have underestimated rates of secondary bacterial infection. We could not retrospectively assess appropriateness of antibiotics according to local guidelines. The long time-interval between study period and reporting reflects the current difficulties of collecting routine clinical data. Although this study was based on data from patients in NHS hospitals in the UK, the challenges faced by other healthcare systems during the first wave COVID-19 pandemic were similar with respect to the research question that we studied and therefore, these results are likely to be relevant to other healthcare systems. However, overgeneralization and assumption of causation should be avoided. Only a future randomized controlled trial (RCT) would overcome these caveats. The primary aim of the study was to measure the difference in the number of days of early antibiotic treatment between patients with COVID-19 who did/did not have baseline PCT testing. A limitation of the study is that the effects of testing of repeated PCT were not studied. Baseline PCT testing was specifically chosen because it presents a straightforward and implementable protocol for hospitals.

### Conclusions

Baseline PCT testing was associated with a statistically significant reduction in antibiotic prescribing in hospitalized patients with COVID-19, indicating that PCT may have been an effective antimicrobial stewardship tool during the first wave of the pandemic. PCT testing appeared to be safe having no measurable impact on mortality or LOS, pending the results of RCTs, and considering the likely cost-effectiveness of PCT in this context (reported in the accompanying article^26^), it seems reasonable for centres who adopted baseline PCT as an antimicrobial stewardship measure to continue to do so. Our study highlights the need for embedded, rapid evaluations of infection diagnostics in the NHS so that even in challenging circumstances, introduction into clinical practice is supported by evidence for clinical utility.

## Supplementary Material

dkae246_Supplementary_Data

## Transparency declarations

J.A.T.S. has received research funding from MRC, EPSRC, NIHR, lumix diagnostics, and Jon Moulton Charity Trust in relation to development of infection diagnostics and is a council member of the British Society for Antimicrobial Chemotherapy (BSAC). J.A.T.S. is co-Investigator (co-I) for PROTECT (NIHR HTA 156664). J.A.T.S. has received honoraria from Medtronic and Tillotts Pharma. M.A. has received honoraria payments for lectures and research from Shionogi, Merck and Pfizer and received research funding from The Showering Fund. L.B.-H. is co-I for PRONTO (NIHR HTA 17/136/13) and BATCH (NIHR HTA 15/188/42). P.D. is Chief Investigator (CI) for ADAPT-Sepsis (NIHR HTA 15/99/02) and co-I for PROTECT (NIHR HTA 156664). J.E. is co-I for PROTECT (NIHR HTA 156664). R.H. is a committee member for the Pharmacy Infection Network (UK Clinical Pharmacy Association) and was a member of the Public Engagement and Patient support committee for Antibiotic Research UK during the time of the study. T.P.H. is CI for RISC-Sepsis (NIHR EME 128374) and CI for SHORTER (NIHR HTA 134101). S.H. has received funding for NIHR Health Protection Research Units (HPRU). D.H. has received institutional grant payments from ESRC, personal consulting fees from Organisation for Economic Co-operation and Development and United Nations Asia-Pacific Region and honoraria payments for lectures and research from University of Lucerne. M.J.L. is co-Chief Investigator (co-CI) for DURATION UTI (NIHR HTA 134854) and co-I for PRONTO (NIHR HTA 17/136/13), the ARC Hospital programme (NIHR PGfAR RP-PG-0514-20015) and SNAP (NIHR HTA 133719).

I.J.M. is co-I for RISC-Sepsis (NIHR EME 128374). D.G.P. is British Infection Association President. N.P. has received honoraria payments for lectures from ThermoFisher. D.S. is co-I for RISC-Sepsis (NIHR EME 128374). T.S. has received fees from ThermoFisher Ltd in relation to PCT testing in the ICU and is CI for VAMp-SEPSIS (HCRW 1951/2023) and co-I for EXTEND (NIHR HTA 131784). E.T.-J. is co-I for PRONTO (NIHR HTA 17/136/13), BATCH (NIHR HTA 15/188/42) and PROTECT (NIHR HTA 156664). S.T. is co-CI for PRONTO (NIHR HTA 17/136/13) and co-I for PROTECT (NIHR HTA 156664). S.T. has also received funding from INNOVATE UK (Joint award through SME pipeline, LUHFT/LSTM/Gencoa Ltd). E.D.C. is CI for BATCH (NIHR HTA 15/188/42), co-CI for PROTECT (NIHR HTA 156664), co-I for PRONTO (NIHR HTA 17/136/13), a former member of the NICE Diagnostic advisory committee (2014–2020) and NICE Sepsis guideline development committee (2014–2016). E.D.C. has been a Scientific Advisory Board member for ThermoFisher and bioMerieux, but never received any personal honoraria, as fees were paid to the University of Liverpool. P.P. is co-I for PRONTO (NIHR HTA 17/136/13) and co-CI for PROTECT (NIHR HTA 155664). All other authors declare no competing interests.

## Data sharing

This work was done under the ‘COPI Notice’ issued under Regulation 3(4) and the corresponding transition to Section 5 of the Health Service (Control of Patient Information) Regulations 2002 to allow processing of confidential patient information without consent. As a result, we cannot make the underlying dataset publicly available for ethical and legal reasons. However, all the data used for this analysis are held as aggregated data by the Centre for Trials Research at Cardiff University. Requests for access to relevant anonymized data should be submitted to the Centre for Trials Research at PEACH@cardiff.ac.uk. It should be noted that within the remits of Condition 1 of the Health Service Regulations, sensitive information cannot be shared. All data releases are subject to receipt of a signed sample and data application form and internal assessment and approval for data release.

The statistical analysis plan has been published and is publicly available as supplementary information to the PEACH protocol paper (doi.org/10.3390/mps5060095)

## Supplementary data

Figures S1 to S7, Tables S1 to S25, are available as Supplementary data at *JAC* Online.
