# Supplementary material for: A retrospective propensity-score-matched cohort study of the impact of procalcitonin testing on antibiotic use in hospitalized patients during the first wave of COVID-19
Source: J Antimicrob Chemother. 2024 Sep 9;79(11):2792–800. doi: 10.1093/jac/dkae246 (PMC11531821; doi:10.1093/jac/dkae246)
Supplement: dkae246_Supplementary_Data [file dkae246_supplementary_data.docx]

**A retrospective propensity-score-matched cohort study of the impact of procalcitonin testing on antibiotic use in hospitalised patients during the first wave of COVID-19 (Sandoe *et al.):* Supplementary Information**

**Contents**

Part 1 Results of secondary (ATU) analysis

Part 2 Supplemental tables and figures

2.1 Propensity score matching process

2.2 Descriptive statistics

2.3 Primary outcome analysis

2.4 Secondary outcome analysis

2.4.1 Association between baseline PCT testing and antibiotic prescribing (total/late)

2.4.2 Association between baseline PCT testing and mortality at 30 and 60 days

2.4.3 Association between baseline PCT testing and length of stay (hospital and ICU)

2.4.4 Association between baseline PCT testing and resistant secondary bacterial infection

2.5 Subgroup analysis: To investigate if the effect of baseline PCT testing on the primary outcome differed dependent on ICU admission at baseline

Part 3 Additional methods/statistics references

Part 4 PEACH study team

**Abbreviations**

ATT: average effect of baseline PCT testing on the tested population

ATU: average effect of baseline PCT testing on the untested population

CI: confidence interval

ICU: intensive care unit

PCT: procalcitonin

SD: standard deviation

SE: standard error

**Part 1 Results of secondary (ATU) analysis**

The estimated effect of the secondary (ATU) analysis was a decrease by 0.30 days (SE = 0.10, 95% CI [-0.11, -0.49], p = 0.002), indicating that the average estimated effect of PCT testing at baseline for people with similar characteristics to those who did not receive it, would be a decrease in the duration of early antibiotics by 0.30 days.

**Part 2 Supplemental tables and figures**

**2.1 Propensity score matching process**

**Figure S1: Balance diagnostics for the propensity score matched data with respect to the primary (ATT) analysis (n= 5960).**

**Figure S2: Balance diagnostics for the propensity score matched data with respect to the secondary (ATU) analysis (n=1818).**

**Part 2.2 Descriptive statistics**

|  | **Frequency (%)** |
| --- | --- |
| **Age category** | |
| 16-49 | 684 (11.2) |
| 50-59 | 695 (11.4) |
| 60-69 | 894 (14.7) |
| 70-79 | 1427 (23.4) |
| >80 | 2387 (39.2) |
| Unknown | 2 (0.0) |
| **Sex** | |
| Female | 2706 (44.4) |
| Male | 3733 (55.4) |
| Unknown | 10 (0.2) |
| **Ethnicity** | |
| White | 4599 (77.2) |
| Mixed | 47 (0.8) |
| Asian | 247 (4.1) |
| Black | 152 (2.5) |
| Other | 246 (4.0) |
| Unknown | 698 (11.5) |
| **Smoking status** | |
| No | 2532 (41.6) |
| Yes | 278 (4.6) |
| Ex-smoker | 1587 (26.1) |
| Unknown | 1692 (27.8) |
| **ICU admission at baseline** | |
| No | 5634 (92.5) |
| Yes | 399 (6.6) |
| Unknown | 56 (0.9) |
| **Has the patient died (as of when the data were collected and input in the study database)** | |
| No | 3375 (55.4) |
| Yes | 2680 (44.0) |
| Unknown | 34 (0.6) |
| **Treatment: dexamethasone** | |
| No | 5826 (95.7) |
| Yes | 230 (3.8) |
| Unknown | 33 (0.5) |
| **Treatment: tocilizumab** | |
| No | 6056 (99.5) |
| Yes | 6 (0.1) |
| Unknown | 27 (0.4) |
| **Treatment: remdesivir** | |
| No | 6009 (98.7) |
| Yes | 50 (0.8) |
| Unknown | 30 (0.5) |

**Table S1: Patient characteristics for the whole sample set passing quality control (n=6089).**

| **Comorbidities** | **Frequency (%)** |
| --- | --- |
| **Asthma** | |
| Yes | 747 (12.3) |
| No | 4195 (68.9) |
| Unknown | 1147 (18.8) |
| **Atrial fibrillation** | |
| Yes | 957 (15.7) |
| No | 3980 (65.4) |
| Unknown | 1152 (18.9) |
| **Cancer** | |
| Yes | 842 (13.8) |
| No | 4098 (67.3) |
| Unknown | 1149 (18.9) |
| **Cardiovascular** | |
| Yes | 613 (10.1) |
| No | 4327 (71.1) |
| Unknown | 1149 (18.8) |
| **Chronic kidney disease stage 1-2** | |
| Yes | 255 (4.2) |
| No | 4672 (76.7) |
| Unknown | 1162 (19.1) |
| **Chronic kidney disease stage 3-5** | |
| Yes | 828 (13.6) |
| No | 4098 (67.3) |
| Unknown | 1163 (19.1) |
| **Chronic neurological conditions** | |
| Yes | 309 (5.1) |
| No | 4633 (76.1) |
| Unknown | 1147 (18.8) |
| **Connective tissue disease** | |
| Yes | 44 (0.7) |
| No | 4897 (80.4) |
| Unknown | 1148 (18.9) |
| **COPD** | |
| Yes | 789 (13.0) |
| No | 4150 (68.2) |
| Unknown | 1150 (18.8) |
| **Coronary heart disease** | |
| Yes | 848 (13.9) |
| No | 4092 (67.2) |
| Unknown | 1149 (18.9) |
| **Dementia** | |
| Yes | 856 (14.1) |
| No | 4087 (67.1) |
| Unknown | 1146 (18.8) |
| **Depression** | |
| Yes | 603 (9.9) |
| No | 4339 (71.3) |
| Unknown | 1147 (18.8) |
| **Diabetes** | |
| Yes | 1605 (26.4) |
| No | 3336 (54.8) |
| Unknown | 1148 (18.8) |
| **Epilepsy** | |
| Yes | 215 (3.5) |
| No | 4727 (77.6) |
| Unknown | 1147 (18.8) |
| **Heart failure** | |
| Yes | 539 (8.9) |
| No | 4404 (72.3) |
| Unknown | 1146 (18.8) |
| **HIV/AIDS** | |
| Yes | 17 (0.3) |
| No | 4922 (80.8) |
| Unknown | 1150 (18.9) |
| **Hypertension** | |
| Yes | 2392 (39.3) |
| No | 2549 (41.9) |
| Unknown | 1148 (18.8) |
| **Learning disability** | |
| Yes | 105 (1.7) |
| No | 4837 (79.4) |
| Unknown | 1147 (18.8) |
| **Mental health** | |
| Yes | 312 (5.1) |
| No | 4628 (76.0) |
| Unknown | 1149 (18.9) |
| **Mild-to-severe liver disease** | |
| Yes | 136 (2.2) |
| No | 4805 (78.9) |
| Unknown | 1148 (18.8) |
| **Obesity (no BMI value)** | |
| Yes | 255 (4.2) |
| No | 4644 (76.3) |
| Unknown | 1190 (19.5) |
| **Obesity (with BMI value)** | |
| Yes | 240 (3.9) |
| No | 4644 (76.3) |
| Unknown | 1205 (19.8) |
| **Osteoporosis** | |
| Yes | 282 (4.6) |
| No | 4660 (76.5) |
| Unknown | 1147 (18.8) |
| **Palliative care** | |
| Yes | 181 (3.0) |
| No | 4760 (78.2) |
| Unknown | 1148 (18.8) |
| **Peripheral arterial disease** | |
| Yes | 126 (2.1) |
| No | 4814 (79.1) |
| Unknown | 1149 (18.9) |
| **Rheumatoid arthritis** | |
| Yes | 125 (2.0) |
| No | 4814 (79.1) |
| Unknown | 1150 (18.9) |
| **Stroke (non-transient ischaemic attack)** | |
| Yes | 360 (5.9) |
| No | 4580 (75.2) |
| Unknown | 1149 (18.9) |
| **Stroke (transient ischaemic attack)** | |
| Yes | 425 (7.0) |
| No | 4512 (74.1) |
| Unknown | 1149 (18.9) |

**Table S2: Comorbidities for the whole sample set passing quality control (n=6089).**

| **Site** | **Frequency (%)** |
| --- | --- |
| Leeds Teaching Hospitals NHS Trust | 885 (14.9) |
| Sheffield Teaching Hospital NHS Foundation Trust | 860 (14.4) |
| Mid Yorkshire Hospitals NHS Trust | 848 (14.2) |
| Nottingham University Hospitals NHS Trust | 795 (13.3) |
| Liverpool University Hospitals NHS Foundation Trust | 582 (9.8) |
| University Hospital Sussex NHS Foundation Trust | 443 (7.4) |
| North Bristol NHS Trust | 388 (6.5) |
| Newcastle-upon-Tyne Hospitals NHS Foundation Trust | 380 (6.4) |
| Aneurin Bevan University Health Board | 340 (5.7) |
| Salford Royal NHS Foundation Trust | 287 (4.8) |
| Royal Cornwall Hospitals NHS Trust | 152 (2.6) |
| Total | 5960 (100.0) |

**Table S3: Recruitment for the 11 hospital Trusts and Health Boards, unmatched (unweighted) quality-controlled data set.**

| **Primary data sources** | **Frequency (%)** |
| --- | --- |
| Full electronic health records (from prescribing system, chemistry lab database, microbiology lab database and radiology database) | 1869 (31.4) |
| Full electronic health records integrated with primary care records | 1737 (29.1) |
| Hybrid process (paper and electronic records) | 1490 (25.0) |
| Hybrid process with no primary care records | 836 (14.0) |
| Full paper records | 27 (0.45) |
| Missing data source | 1 (0.02) |
| Total | 5960 (100) |

**Table S4: Primary data sources for unmatched (unweighted) data set.**

|  | **Frequency (%)** |
| --- | --- |
| COVID-19 test before hospital admission | 225 (3.8) |
| Community onset (0 -2 days) | 4346 (72.9) |
| Indeterminate onset (3-7 days) | 457 (7.7) |
| Probable nosocomial onset (8-14 days) | 419 (7.0) |
| Definite nosocomial onset (>15 days) | 513 (8.6) |
| Total | 5960 (100) |

**Table S5: Onset of COVID-19: community or hospital, unmatched (unweighted) data set.**

|  | **PCT at baseline, unmatched sample (total number = 2818)** | | **PCT at baseline, matched sample (total number = 2818)** | |
| --- | --- | --- | --- | --- |
|  | **No** | **Yes** | **No** | **Yes** |
|  | **Frequency (%)** | **Frequency (%)** | **Frequency (%)** | **Frequency (%)** |
| **Age category** | | | | |
| 16-49 | 165 (12.6) | 182 (12.0) | 165 (12.6) | 175.6 (11.6) |
| 50-59 | 154 (11.8) | 222 (14.7) | 154 (11.8) | 209.8 (13.9) |
| 60-69 | 211 (16.2) | 244 (16.1) | 211 (16.2) | 232.5 (15.4) |
| 70-79 | 303 (23.2) | 337 (22.3) | 303 (23.2) | 341.4 (22.6) |
| >80 | 472 (36.2) | 528 (35.0) | 472 (36.2) | 553.6 (36.6) |
| **Sex** | | | | |
| Female | 566 (43.4) | 653 (43.2) | 566 (43.4) | 654.5 (43.3) |
| Male | 737 (56.5) | 858 (56.7) | 737 (56.5) | 856.2 (56.6) |
| Unknown | 2 (0.2) | 2 (0.1) | 2 (0.2) | 2.3 (0.2) |
| **Ethnicity** | | | | |
| White | 1041 (79.8) | 1206 (79.7) | 1041 (79.8) | 1211.0 (80.0) |
| Mixed | 10 (0.8) | 11 (0.7) | 10 (0.8) | 12.2 (0.8) |
| Asian | 63 (4.8) | 98 (6.5) | 63 (4.8) | 86.4 (5.7) |
| Black | 39 (3.0) | 57 (3.8) | 39 (3.0) | 53.3 (3.5) |
| Other | 47 (3.6) | 52 (3.4) | 47 (3.6) | 52.2 (3.5) |
| Unknown | 105 (8.1) | 89 (5.9) | 105 (8.1) | 98.0 (6.5) |
| **Smoking status** | | | | |
| No | 482 (36.9) | 604 (39.9) | 482 (36.9) | 580.9 (38.4) |
| Yes | 49 (3.8) | 62 (4.1) | 49 (3.8) | 60.9 (4.0) |
| Ex-smoker | 291 (22.3) | 346 (22.9) | 291 (22.3) | 343.2 (22.7) |
| Unknown | 483 (37.0) | 501 (33.1) | 483 (37.0) | 528.1 (34.9) |
| **ICU admission at baseline** | | | | |
| No | 1169 (89.6) | 1316 (87.0) | 1169 (89.6) | 1342.0 (89.6) |
| Yes | 123 (9.4) | 184 (12.2) | 123 (9.4) | 160.0 (10.6) |
| Unknown | 13 (1.0) | 13 (0.9) | 13 (1.0) | 11.0 (0.7) |
| **Has the patient died (as of when the data were collected and input in the study database)** | | | | |
| No | 696 (53.3) | 898 (59.4) | 696 (53.3) | 891.0 (58.9) |
| Yes | 600 (46.0) | 609 (40.3) | 600 (46.0) | 616.2 (40.7) |
| Unknown | 9 (0.7) | 6 (0.4) | 9 (0.7) | 5.8 (0.4) |

**Table S6: Patient characteristics according to whether a PCT test was done at baseline or not, showing both propensity score matched (weighted) and unmatched (unweighted) data with respect to the ATU.**

|  | **Unmatched data**  **(total number = 5960)** | **Matched data based on ATT**  **(total number = 5960)** |
| --- | --- | --- |
| **Number of comorbidities** | **Frequency (%)** | **Frequency (%)** |
| **0** | 1134 (19.0) | 1340.7 (22.5) |
| **1** | 971 (16.3) | 1334.2 (22.4) |
| **2** | 1,151 (19.3) | 1214.2 (20.4) |
| **3** | 1,036 (17.4) | 898.6 (10.2) |
| **4** | 781 (13.1) | 609.4 (9.7) |
| **5** | 436 (7.3) | 296.0 (5.0) |
| **6** | 244 (4.1) | 152.7 (2.6) |
| **7** | 120 (2.0) | 69.3 (1.2) |
| **8** | 45 (0.8) | 26.5 (0.4) |
| **9** | 25 (0.4) | 12.1 (0.2) |
| **10** | 10 (0.2) | 3.2 (0.1) |
| **11** | 4 (0.1) | 2.5 (0.0) |
| **12** | 1 (0.0) | 0.1 (0.0) |
| **14** | 1 (0.0) | 1.0 (0.0) |
| **19** | 1 (0.0) | 0.04 (0.0) |

**Table S7: Number of comorbidities per person for the unmatched and matched data (based on primary ATT analysis).**

|  | **Unmatched data (total number = 5960)** | **Matched data based on ATT (total number = 5960)** |
| --- | --- | --- |
|  |  |  |
| **Comorbidities** | **Frequency (%)** | **Frequency (%)** |
|  |  |  |
| **Asthma** | | |
| Yes | 727 (12.2) | 607.9 (10.2) |
| No | 4122 (69.2) | 4039.9 (67.8) |
| Unknown | 1111 (18.6) | 1312.9 (22.0) |
| **Atrial fibrillation** | | |
| Yes | 947 (15.9) | 827.9 (13.9) |
| No | 3987 (65.4) | 3813.4 (64.0) |
| Unknown | 1116 (18.7) | 1318.7 (22.6) |
| **Cancer** | | |
| Yes | 828 (13.9) | 683.9 (11.5) |
| No | 4019 (67.4) | 3962.0 (66.5) |
| Unknown | 1114 (18.7) | 1314.0 (22.0) |
| **Cardiovascular** | | |
| Yes | 603 (10.1) | 441.0 (7.4) |
| No | 4244 (71.2) | 4200.4 (70.5) |
| Unknown | 1113 (18.6) | 1318.6 (22.1) |
| **Chronic kidney disease stage 1-2** | | |
| Yes | 248 (4.2) | 197.6 (3.3) |
| No | 4586 (77.0) | 4422.9 (74.2) |
| Unknown | 1113 (18.7) | 1339.4 (22.5) |
| **Chronic kidney disease stage 3-5** | | |
| Yes | 816 (13.7) | 638.7 (10.7) |
| No | 4017 (67.4) | 3978.2 (66.8) |
| Unknown | 1112 (18.6) | 1343.1 (22.5) |
| **Chronic neurological conditions** | | |
| Yes | 303 (5.1) | 253.4 (4.3) |
| No | 4546 (76.3) | 4393.7 (73.7) |
| Unknown | 1111 (18.6) | 1312.9 (22.0) |
| **Connective tissue disease** | | |
| Yes | 43 (0.7) | 24.0 (0.4) |
| No | 4805 (80.6) | 4622.8 (77.6) |
| Unknown | 1112 (18.6) | 1313.2 (22.0) |
| **COPD** | | |
| Yes | 780 (13.1) | 591.6 (9.9) |
| No | 4066 (68.2) | 4053.9 (68.0) |
| Unknown | 1114(18.7) | 1314.4 (22.1) |
| **Coronary heart disease** | | |
| Yes | 839 (14.1) | 665.9 (11.2) |
| No | 4008 (67.3) | 3979.2 (66.8) |
| Unknown | 1113 (18.7) | 1314.8 (22.1) |
| **Dementia** | | |
| Yes | 842 (14.1) | 645.3 (10.8) |
| No | 4007 (67.2) | 4001.7 (67.1) |
| Unknown | 1111 (18.6) | 1312.9 (22.0) |
| **Depression** | | |
| Yes | 589 (9.9) | 528.2 (8.9) |
| No | 4260 (71.5) | 4118.9 (69.1) |
| Unknown | 1111 (18.6) | 1369.1 (23.0) |
| **Diabetes** | | |
| Yes | 1578 (26.5) | 1455.2 (24.4) |
| No | 3270 (54.9) | 3189.0 (53.5) |
| Unknown | 1113 (18.6) | 1315.8 (22.1) |
| **Epilepsy** | | |
| Yes | 215 (3.6) | 151.4 (2.5) |
| No | 4634 (77.8) | 4495.4 (75.4) |
| Unknown | 1111 (18.6) | 1312.9 (22.0) |
| **Heart failure** | | |
| Yes | 528 (8.9) | 396.7 (6.7) |
| No | 4321 (72.5) | 4250.4 (71.3) |
| Unknown | 1111 (18.6) | 1312.9 (22.0) |
| **HIV/AIDS** | | |
| Yes | 17 (0.3) | 12.9 (0.2) |
| No | 4829 (81.0) | 4633.4 (77.7) |
| Unknown | 1114 (18.7) | 1313.7 (22.0) |
| **Hypertension** | | |
| Yes | 2351 (39.5) | 2146.1 (36.0) |
| No | 2496 (41.9) | 2500.2 (41.9) |
| Unknown | 1111 (18.6) | 1313.7 (22.0) |
| **Learning disability** | | |
| Yes | 104 (1.7) | 100.2 (1.7) |
| No | 4745 (79.6) | 4546.8 (76.3) |
| Unknown | 1111 (18.6) | 1312.9 (22.0) |
| **Mental health** | | |
| Yes | 305 (5.1) | 233.2 (3.9) |
| No | 4542 (76.2) | 4413.6 (74.1) |
| Unknown | 1111 (18.6) | 1313.3 (22.0) |
| **Mild-to-severe liver disease** | | |
| Yes | 135 (2.3) | 86.6 (1.5) |
| No | 4713 (79.1) | 4560.3 (76.5) |
| Unknown | 1112 (18.6) | 1313.2 (22.0) |
| **Obesity (no BMI value)** | | |
| Yes | 249 (4.2) | 223.0 (3.7) |
| No | 4557 (76.5) | 4389.4 (73.7) |
| Unknown | 1151 (19.3) | 1347.7 (22.6) |
| **Obesity (with BMI value)** | | |
| Yes | 235 (3.9) | 191.6 (3.2) |
| No | 4555 (76.4) | 4399.5 (73.8) |
| Unknown | 1159 (19.6) | 1368.9 (23.0) |
| **Osteoporosis** | | |
| Yes | 277 (4.7) | 204.5 (3.4) |
| No | 4572 (76.7) | 4442.6 (74.5) |
| Unknown | 1111 (18.6) | 1312.9 (22.0) |
| **Palliative care** | | |
| Yes | 178 (3.0) | 120.2 (2.0) |
| No | 4670 (78.4) | 4526.8 (76.0) |
| Unknown | 1113 (18.7) | 1313.0 (22.0) |
| **Peripheral arterial disease** | | |
| Yes | 124 (2.1) | 69.3 (1.2) |
| No | 4723 (79.2) | 4577.4 (76.8) |
| Unknown | 1113 (18.7) | 1313.3 (22.0) |
| **Rheumatoid arthritis** | | |
| Yes | 124 (2.1) | 90.6 (1.5) |
| No | 4722 (79.2) | 4554.5 (76.4) |
| Unknown | 1114 (18.7) | 1315.0 (22.1) |
| **Stroke (non-transient ischaemic attack)** | | |
| Yes | 355 (6.0) | 269.5 (4.5) |
| No | 4491 (75.4) | 4377.0 (73.4) |
| Unknown | 1114 (18.7) | 1313.5 (22.0) |
| **Stroke (transient ischaemic attack)** | | |
| Yes | 423 (7.1) | 312.1 (5.2) |
| No | 4421 (74.2) | 4334.1 (72.7) |
| Unknown | 1116 (18.7) | 1313.9 (22.0) |

**Table S8: Comorbidities for the unmatched and matched data (based on ATT).**

**2.3 Primary outcome analysis**

**Figure S3: Histogram of days of primary outcome (early antibiotics, within first 7 days) according to whether a PCT test was done at baseline or not (n=2818).**

Using propensity score matching based on the ATU.

**2.4 Secondary outcome analysis**

**2.4.1 Association between baseline PCT testing and antibiotic therapy (total/late)**

**Figure S4: Histogram of total antibiotic days according to whether PCT test was done at baseline or not (n=5960).** Data are matched according to propensity score matching based on the ATT. The data with respect to the total days on antibiotics were truncated at 30 days for the purpose of presenting the figure. The mean number of total days on antibiotics was 6.6 (median 5, SD 7.2, range 0-97 days).

**Figure S5: Histogram of late antibiotic days according to whether PCT test was done at baseline or not (n=5960).** Data are matched according to propensity score matching based on the ATT. The data with respect to the late days on antibiotics were truncated at 30 days for the purposes of presenting the figure. The mean number of late days on antibiotics was 2.6 (median 0, SD 6.1, range 0-92 days).

Subgroup analysis: To investigate if the number of PCT tests was associated with the duration of total antibiotic treatment, and the number of PCT tests done at any time was explored.

|  | **Estimate** | **SE** | **t-value** | **p-value** |
| --- | --- | --- | --- | --- |
| (Intercept) | 5.49 | 0.11 | 49.90 | <0.001 |
| PCT test done at baseline | -5.18 | 0.27 | -19.05 | <0.001 |
| 1 PCT test at any time | 3.58 | 0.27 | 13.26 | <0.001 |
| 2 or more PCT tests at any time | 9.78 | 0.28 | 34.55 | <0.001 |

**Table S9: The effect size estimates of the impact of the number of PCT tests on total days on antibiotic treatment using linear regression and the primary (ATT) matched sample (n=5960).**

|  | **Estimate** | **SE** | **t-value** | **p-value** |
| --- | --- | --- | --- | --- |
| (Intercept) | 1.58 | 0.09 | 16.85 | <0.001 |
| PCT test done at baseline | -4.17 | 0.23 | -17.98 | <0.001 |
| 1 PCT test at any time | 3.18 | 0.22 | 13.85 | <0.001 |
| 2 or more PCT tests at any time | 8.36 | 0.24 | 34.63 | <0.001 |

**Table S10: The effect size estimates of the impact of the number of PCT tests on late days on antibiotic treatment using linear regression and the primary (ATT) matched sample (n=5960).**

**2.4.2 Association between baseline PCT testing and mortality at 30 and 60 days**

|  | **PCT at baseline, unmatched sample (total number = 5960)** | | **PCT at baseline, matched ATT sample (total number = 5960)** | |
| --- | --- | --- | --- | --- |
|  | **No** | **Yes** | **No** | **Yes** |
|  | **Frequency (%)** | **Frequency (%)** | **Frequency (%)** | **Frequency (%)** |
| **Mortality at 30 days** | | | | |
| No | 2993 (67.8) | 1036 (66.9) | 2948.4 (66.8) | 1036 (66.9) |
| Yes | 1419 (32.2) | 512 (33.1) | 1463.6 (33.2) | 512 (33.1) |
| **Mortality at 60 days** | | | | |
| No | 2872 (65.1) | 996 (64.3) | 2827.4 (64.1) | 996 (64.3) |
| Yes | 1540 (34.9) | 552 (35.7) | 1584.6 (35.9) | 552 (35.7) |

**Table S11: Mortality at 30 and 60 days, in propensity score matched ATT sample (n=5960).**

The results with respect to the matched data for ATT for mortality at 30 days, are presented in the table below:

|  | **Estimate** | **SE** | **z-value** | **p-value** |
| --- | --- | --- | --- | --- |
| (Intercept) | -0.70 | 0.03 | -21.90 | <0.001 |
| PCT test done at baseline | -0.004 | 0.06 | -0.07 | 0.944 |

**Table S12: Effect size estimates by the logistic regression for the relationship between baseline PCT and mortality at 30 days using propensity score matched sample (ATT, n=5960).**

The results with respect to the matched data for ATT for mortality at 60 days, are presented in the table below:

|  | **Estimate** | **SE** | **z-value** | **p-value** |
| --- | --- | --- | --- | --- |
| (Intercept) | -0.58 | 0.03 | -18.45 | <0.001 |
| PCT test done at baseline | -0.01 | 0.06 | -0.18 | 0.857 |

**Table S13: Effect size estimates by the logistic regression for the relationship between baseline PCT and mortality at 60 days using propensity score matched sample (ATT) (n=5960).**

**2.4.3 Association between baseline PCT testing and length of stay (hospital and ICU)**

|  | **PCT at baseline, no** | | | | **PCT at baseline, yes** | | | |
| --- | --- | --- | --- | --- | --- | --- | --- | --- |
|  | Number (unweighted) | Number (weighted) | Mean | SD | Number (unweighted) | Number (weighted) | Mean | SD |
| Inpatient stay (days) | 4354 | 4378.66 | 16.36 | 31.45 | 1528 | 1528 | 13.41 | 16.94 |

**Table S14: Descriptive statistics for days of hospital inpatient stay by PCT test done at baseline or not; matched sample (ATT, n=5882).**

The results with respect to the matched data for ATT for the log-transformed data for the length of stay, are presented in the table below. The length of stay variable had to be logarithmically transformed due to skewness and outliers in the data.

|  | **Estimate** | **SE** | **t-value** | **p-value** |
| --- | --- | --- | --- | --- |
| (Intercept) | 2.01 | 0.02 | 99.52 | <0.001 |
| PCT test done at baseline | 0.07 | 0.04 | 1.71 | 0.088 |

**Table S15: Effect size estimates by the linear regression for the relationship between baseline PCT and hospital inpatient stay using propensity score matched sample (ATT, n=5882).**

The spread of the logarithmically transformed length of stay in the propensity score matched ATT data, broken down by baseline PCT, is presented in **Figure S6.**

**Figure S6: Histogram of logarithmically transformed length of hospital stay according to whether PCT test was done at baseline or not (n=5882).**

Data are matched according to propensity score matching based on the ATT.

Association between baseline PCT and ICU length of stay (days >48h after Day 1)

|  | **PCT at baseline: Not done, ATT matched sample** | | | | **PCT at baseline: Done, ATT matched sample** | | | |
| --- | --- | --- | --- | --- | --- | --- | --- | --- |
|  | Number (unweighted) | Number (weighted) | Mean | SD | Number (unweighted) | Number (weighted) | Mean | SD |
| ICU stay (days) | 4378 | 4373.83 | 2.92 | 9.88 | 1538 | 1538 | 2.32 | 7.59 |

**Table S16: Descriptive statistics for ICU length of stay >48h after Day 1 by PCT test done at baseline or not, using propensity score matched sample (ATT, n=5916).** The “ICU stay” variable was missing data for 44 individuals, hence analysis on n=5916

|  | **PCT at baseline: Not done, ATT matched sample** | | | | **PCT at baseline: Done, ATT matched sample** | | | |
| --- | --- | --- | --- | --- | --- | --- | --- | --- |
|  | Number (unweighted) | Number (weighted) | Mean | SD | Number (unweighted) | Number (weighted) | Mean | SD |
| ICU stay (days) | 279 | 650.91 | 19.60 | 18.18 | 266 | 266 | 13.41 | 13.61 |

**Table S17: Descriptive statistics for ICU stay >48h after Day 1 by PCT test done at baseline or not using propensity score matched sample (ATT, n=5916). Individuals with 0 days in ICU have been excluded.**

The results with respect to the matched data for ATT for the log-transformed data for the ICU length of stay (>48 hours after Day 1), are presented in the table below. The length of ICU stay variable was logarithmically transformed because of skewness and outliers in the data.

|  | **Estimate** | **SE** | **t-value** | **p-value** |
| --- | --- | --- | --- | --- |
| (Intercept) | -2.18 | 0.03 | -72.58 | <0.001 |
| PCT test done at baseline | 0.06 | 0.06 | 1.09 | 0.274 |

**Table S18: Effect size estimates by the linear regression for the relationship between baseline PCT and logarithmically transformed ICU length of stay using propensity score matched sample (ATT, n=5916).**

**Figure S7: Histogram of logarithmically transformed ICU length of stay (>48 hours after Day 1) according to whether PCT test was done at baseline or not.**

Data are matched according to propensity score matching based on the ATT (n=5916).

**2.4.4 Association between baseline PCT testing and resistant secondary bacterial infection**

| **Secondary bacterial infection** | **PCT at baseline**  **Unmatched sample**  **(total number = 5960)** | | **PCT at baseline**  **ATT matched sample**  **(total number = 5960)** | |
| --- | --- | --- | --- | --- |
|  | No | Yes | No | Yes |
|  | Frequency (%) | Frequency (%) | Frequency (%) | Frequency (%) |
| **No** | 4295 (97.4) | 1501 (97.0) | 4129.4 (95.6) | 1501 (97.0) |
| **Yes** | 87 (2.0) | 46 (3.0) | 184.6 (4.2) | 46 (3.0) |
| **Unknown** | 30 (0.7) | 1 (0.1) | 8.0 (0.2) | 1 (0.1) |

**Table S19: Descriptive statistics for secondary bacterial infection by PCT test done at baseline or not.** Resistance to antibiotics is presented in Table S20.

|  | **PCT at baseline**  **Unmatched sample**  **(total number = 5960)** | | **PCT at baseline**  **ATT matched sample**  **(total number = 5960)** | |
| --- | --- | --- | --- | --- |
|  | **No** | **Yes** | **No** | **Yes** |
|  | **Frequency (%)** | **Frequency (%)** | **Frequency (%)** | **Frequency (%)** |
| **Not resistant** | 30 (0.7) | 9 (0.6) | 67.6 (1.5) | 9 (0.6) |
| **Resistant to 1** | 16 (0.4) | 11 (0.7) | 22.8 (0.5) | 11 (0.7) |
| **Resistant to 2** | 8 (0.2) | 7 (0.5) | 27.6 (0.6) | 7 (0.5) |
| **Resistant to 3 or more** | 15 (0.3) | 13 (0.8) | 31.9 (0.7) | 13 (0.8) |
| **Unknown** | 48 (1.1) | 7 (0.5) | 42.8 (1.0) | 7 (0.5) |
| **Early infection** | 79 (1.8) | 18 (1.2) | 78.5 (1.8) | 18 (1.2) |
| **No infection** | 4126 (5.6) | 1483 (95.8) | 4140.8 (93.9) | 1483 (95.8) |

**Table S20: Descriptive statistics for secondary bacterial infection resistant to 0, 1, 2 or 3 or more antibiotic classes by PCT test done at baseline or not.**

Results with respect to the matched data for ATT for resistant to 3 or more, 2 or 1 antibiotics, are presented in the tables below:

|  | **Estimate** | **SE** | **z-value** | **p-value** |
| --- | --- | --- | --- | --- |
| (Intercept) | -4.92 | 0.18 | -27.69 | <0.001 |
| PCT test done at baseline | 0.15 | 0.33 | 0.46 | 0.646 |

**Table S21: Effect size estimates by the logistic regression for the relationship between baseline PCT and resistance to 3 or more antibiotic classes using propensity score matched sample (ATT, n=5960)**.

|  | **Estimate** | **SE** | **z-value** | **p-value** |
| --- | --- | --- | --- | --- |
| (Intercept) | -4.29 | 0.13 | -32.88 | <0.001 |
| PCT test done at baseline | -0.04 | 0.26 | -0.17 | 0.869 |

**Table S22: Effect size estimates by the logistic regression for the relationship between baseline PCT and resistant to 2 or more antibiotics using propensity score matched sample (ATT, n=5960).**

|  | **Estimate** | **SE** | **z-value** | **p-value** |
| --- | --- | --- | --- | --- |
| (Intercept) | -3.96 | 0.11 | -35.61 | <0.001 |
| PCT test done at baseline | 0.07 | 0.21 | 0.34 | 0.731 |

**Table S23: Effect size estimates by the logistic regression for the relationship between baseline PCT and resistant to 1 or more antibiotics using propensity score matched sample (ATT, n=5960).**

**2.5 Subgroup analysis: To investigate if the effect of baseline PCT on the primary outcome differed dependent on ICU admission at baseline**

|  | **Number (%) of individuals (n=5960), PCT at baseline** | | **Number (%) of individuals (n=5960, matched ATT), PCT at baseline** | | **Mean (SD) number days on early antibiotics/PCT at baseline (n=5960)** | | **Mean (SD) number days on early antibiotics/PCT at baseline, matched ATT (n=5960)** | |
| --- | --- | --- | --- | --- | --- | --- | --- | --- |
|  | **No** | **Yes** | **No** | **Yes** | **No** | **Yes** | **No** | **Yes** |
| ICU at baseline - No | 4193 (95.0) | 1333 (86.1) | 3796.2 (86.0) | 1333 (86.1) | 3.46 (2.69) | 3.55 (2.50) | 3.96 (2.53) | 3.55 (2.50) |
| ICU at baseline - Yes | 184 (4.2) | 200 (12.3) | 571.9 (13.0) | 200 (12.3) | 4.76 (2.41) | 4.31 (2.36) | 5.12 (2.25) | 4.31 (2.36) |
| ICU at baseline - Unknown | 35 (0.8) | 15 (1.0) | 43.9 (1.0) | 15 (1.0) | 2.54 (2.55) | 4.4 (2.44) | 1.62 (2.29) | 4.4 (2.44) |

**Table S24: Descriptive statistics for being in ICU at baseline by PCT test done at baseline or not.**

The results with respect to the matched data for ATT, are presented in the table below:

|  | **Estimate** | **SE** | **t-value** | **p-value** |
| --- | --- | --- | --- | --- |
| (Intercept) | 3.96 | 0.04 | 98.14 | <0.001 |
| PCT test done at baseline | -0.41 | 0.08 | -5.19 | <0.001 |
| ICU at baseline - Yes | 1.16 | 0.11 | 10.37 | <0.001 |
| ICU at baseline - Unknown | -2.33 | 0.38 | -6.18 | <0.001 |
| PCT test done at baseline: ICU at baseline – Yes | -0.40 | 0.22 | -1.82 | 0.068 |
| PCT test done at baseline: ICU at baseline - Unknown | 3.18 | 0.75 | 4.25 | 0.001 |

**Table S25: Effect size estimates by the linear regression for the relationship between baseline PCT and admission to ICU at baseline and days on early antibiotics using propensity score matched sample (ATT, n=5960).**

**Part 3 Additional methods/statistics references**

S1. Singer M, Deutschman CS, Seymour CW, et al. The Third International Consensus Definitions for Sepsis and Septic Shock (Sepsis-3). JAMA 2016; 315(8): 801-10.

S2. Su Y, Tu GW, Ju MJ, et al. Comparison of CRB-65 and quick Sepsis-related Organ Failure Assessment for predicting the need for intensive respiratory or vasopressor support in patients with COVID-19. J Infect 2020; 81(4): 647-79.

S3. Kostakis I, Smith GB, Prytherch D, et al. The performance of the National Early Warning Score and National Early Warning Score 2 in hospitalised patients infected by the severe acute respiratory syndrome coronavirus 2 (SARS-CoV-2). Resuscitation 2021; 159: 150-7.

S4. Gupta RK, Harrison EM, Ho A, et al. Development and validation of the ISARIC 4C deterioration model for adults hospitalised with COVID-19: a prospective cohort study. Lancet Respir Med 2021; 9(4): 349-59.

S5. Greifer N, Stuart EA. Choosing the estimand when matching or weighting in observational studies. arXiv preprint 2021 arXiv:210610577.

S6. VanderWeele TJ, Ding P. Sensitivity analysis in observational research: introducing the E-value. Ann Intern Med 2017; 167(4): 268-74.

S7. R Core Team. R: A language and environment for statistical computing. R Foundation for Statistical Computing, Vienna, Austria. 2022. [https://www.R-project.org](https://www.R-project.org/)

S8. Ho D, Imai K, King G, Stuart EA. MatchIt: Nonparametric Preprocessing for Parametric Causal Inference. J Stat Softw 2011; 42(8): 1-28.

S9. Greifer N. cobalt: Covariate Balance Tables and Plots. R package version 4.5.1. 2023.

S10. Arel-Bundock V. marginaleffects: Predictions, Comparisons, Slopes, Marginal Means, and Hypothesis Tests. R package version 0.13.0. 2023. <https://marginaleffects.com>

S11. Wickham H. ggplot2: Elegant Graphics for Data Analysis. Springer-Verlag, New York. ISBN 978-3-319-24277-4. 2016. <https://ggplot2.tidyverse.org>

Part 4 Appendix of PEACH study team

**Co-Chief Investigators**

Jonathan Sandoe^1,2^

Enitan Carrol^3^

^1^ Department of Microbiology, The General Infirmary at Leeds, Leeds, UK

^2^ Healthcare Associated Infection Group, Leeds Institute of Medical Research, University of Leeds, Leeds, UK

^3^ Department of Clinical Infection, Microbiology and Immunology, Institute of Infection, Veterinary and Ecological Sciences, University of Liverpool, Liverpool, UK

**Coordinating Centre:**

*Study lead*: Emma Thomas-Jones,^1^

*Study Manager*: Joanne Euden,^1^

*Qualitative Researchers*: Lucy Brookes-Howell,^1^ Josie Henley,^2^

*Data Manager*: Wakunyambo Maboshe,^1^

*Co-lead Statistician*: Philip Pallmann,^1^

*Statistician*: Detelina Grozeva,^1^

*Database support*: Marcin Bargiel^1^

*Study Administrator*: Judith Evans^1^

^1^ Centre for Trials Research, College of Biomedical and Life Sciences, Cardiff University, Cardiff, UK

^2^ School of Social Sciences, Cardiff University, King Edward VII Avenue, Cardiff, CF10 3WA

**Research Team**

*Health Economics:* Edward Webb^1^ Rebecca Bestwick,^1^ Daniel Howdon,^1^ Natalie King,^1^ Bethany Shinkins (lead),^1,2^

*Co-lead Statistician*: Robert West,^1^

^1^ Leeds Institute for Health Sciences, University of Leeds, UK

^2^ Division of Health Sciences, University of Warwick, Coventry, UK

**Study Partners:**

*RX Info*: Colin Richman,^1^

*UK Health Security Agency (UKHSA)*: Sarah Gerver,^2^ Russell Hope,^2^ Susan Hopkins,^2^

*Public Health Wales*: Margaret Heginbothom,^3^

*NHS England*: Philip Howard,^4^

^1^ Rx-Info Ltd, Exeter Science Park, Exeter, EX5 2FN, UK

^2^ UK Health Security Agency (UKHSA), UK

^3^ Healthcare Associated Infection, Antimicrobial Resistance and Prescribing Programme, Public Health Wales, UK

^4^ NHS England and NHS Improvement, North-East and Yorkshire Region, UK

**Participating NHS Trusts:**

**Leeds Teaching Hospitals NHS Trust (lead Trust)**

*Principal Investigator*: Jonathan Sandoe,^1,2^

*Research Group (data collection – alphabetical order)* Claire Berry,^3^ Georgina Davis,^3^ Vikki Wilkinson,^3^

^1^ Department of Microbiology, The General Infirmary at Leeds, Leeds, UK

^2^ Healthcare Associated Infection Group, Leeds Institute of Medical Research, University of Leeds, Leeds, UK

^2^ Leeds Teaching Hospitals NHS Trust, Leeds, UK

**Liverpool University Hospitals NHS Foundation Trust**

*Principal Investigator*: Stacy Todd^1^

*Research Group (data collection – alphabetical order):* Eleanor Taylor-Barr,^1^ Mary Brodsky,^1^ Jo Brown^1^ Jenni Burns,^1^ Sharon Glynn,^1^ Alvyda Gureviciute,^1^ Megan Howard,^1^ Jennifer Kirkpatrick,^1^ Hannah Muphy,^1^ Emma Richardson,^1^ Deborah Scanlon,^1^ Claire Small,^1^ Graham Sweeney,^1^ Lisa Williams,^1^

^1^ Liverpool University Hospitals NHS Foundation Trust, Liverpool, UK

**Aneurin Bevan University Health Board**

*Principal Investigator*: Tamas Szakmany^1,2^

*Research Group (data collection – alphabetical order*): Evelyn Baker,^3^ Yusuf Cheema,^3^ Jill Dunhill,^3^ Charlotte Killick,^3^ Charlie King,^3^ Simran Kooner,^3^ Swyn Lewis,^3^ Maxine Nash,^3^ Owen Richardson,^3^ Jemma Tuffney,^3^ Clare Westacott,^3^ Sarah Williams,^3^

^1^ Critical Care Directorate, Aneurin Bevan University Health Board, Cwmbran, UK

^2^ Department of Anaesthesia, Intensive Care and Pain Medicine, Division of Population Medicine, Cardiff University, Cardiff, UK

^3^ Aneurin Bevan University Health Board, Cwmbran, UK

**Sheffield Teaching Hospital NHS Foundation Trust**

*Co-Principal Investigators:* David Partridge,^1^ Helena Parsons,^1^

*Research Group (data collection – alphabetical order*): Kay Cawthron,^1^ Yuen Kiu Tai,^1^ Thomas Newman,^1^ Megan Plowright,^1^ Helen Shulver,^1^ Anna Sivakova,^1^

^1^ Sheffield Teaching Hospitals NHS Foundation Trust, Sheffield, UK

**Royal Cornwall Hospitals NHS Trust**

*Principal Investigator*: Neil Powell^1^

*Research Group (data collection – alphabetical order*): Freddie Ayliffe,^1^ Emma Darke,^1^ Eve Fletcher,^1^ Fiona Hammonds,^1^ Gladys Marquez,^1^ Leanne Welch,^1^

^1^ Royal Cornwall Hospitals NHS Foundation Trust, Truro, UK

**Mid Yorkshire Teaching NHS Trust**

*Principal Investigator*: Stuart Bond^1^

*Research Group (data collection – alphabetical order*): Jade Lee-Milner,^2^

Joseph Spencer,^2^

^1^ Medicines Optimisation and Pharmacy Services, Pindersfield Hospital, Mid Yorkshire Teaching NHS Trust, Wakefield, UK

^2^ Mid Yorkshire Teaching NHS Trust, Wakefield, UK

**North Bristol NHS Trust, Bristol**

*Principal Investigator*: Mahableshwar Albur^1^

*Research Group (data collection – alphabetical order*): Rodrigo Brandao,^1^ Joshua Hrycaiczuk,^1^ Jack Stanley,^1^

^1^ North Bristol NHS Trust, Bristol, UK

**University Hospital Sussex NHS Foundation Trust**

*Principal Investigator*: Martin Llewelyn^1^

*Research Group (data collection – alphabetical order*): Elizabeth Cross,^2^ Daniel Hansen,^2^ Ethan Redmore,^2^ Abigail Whyte,^2^

^1^ Brighton and Sussex Medical School, University of Sussex and University Hospitals Sussex NHS Foundation Trust, Brighton UK

^2^ University Hospitals Sussex NHS Foundation Trust, Brighton, UK

**Newcastle-upon-Tyne Hospitals NHS Foundation Trust**

*Principal Investigators*: Tom Hellyer,^1,2^ Iain McCullagh,^1,2^

*Research Group (data collection – alphabetical order):* Benjamin Brown,^3^ Michele Calabrese,^3^ Cameron Cole,^3^ Jessica DeSousa,^3^ Leigh Dunn,^3^ Stephanie Grieveson,^3^ Arti Gulati,^3^ Elizabeth Issac,^3^ Ruaridh Mackay,^3^ Fatima Simoes,^3^

^1^ Critical Care Department, Royal Victoria Infirmary, The Newcastle-upon-Tyne Hospitals NHS Foundation Trust, Newcastle upon Tyne, UK

^2^ Translational and Clinical Research Institute, Newcastle University, Newcastle upon Tyne, UK

^3^ Newcastle-upon-Tyne Hospitals NHS Foundation Trust, Newcastle upon Tyne, UK

**Salford Royal NHS Foundation Trust**

*Principal Investigator*: Paul Dark^1^

*Research Group (data collection – alphabetical order*): Elena Apatri,^2^ Bethan Charles,^2^ Helen Christensen,^2^ Alice Harvey,^2^ Diane Lomas,^2^ Melanie Taylor,^2^ Vicky Thomas,^2^ Danielle Walker,^2^

^1^ Division of Immunology, Immunity to Infection and Respiratory Medicine, University of Manchester, Manchester, UK

^2^ Salford Royal NHS Foundation Trust, Salford, UK

**Nottingham University Hospitals NHS Trust**

*Principal Investigator*: Dominick Shaw^1^

*Research Group (data collection*): Lucy Howard,^2^ Amelia Joseph,^2^ Saheer Sultan^2^

^1^ Leicester NIHR Biomedical Research Centre and Department of Respiratory Sciences, University of Leicester, Leicester, UK

^2^ Nottingham University Hospitals NHS Trust, Nottingham, UK

**Patient and Public Representatives:**

Chikezie Knox-Macaulay^1^

Margaret Ogden^1^

Graham Prestwich^1^

Ryan Hamilton^2,3^

^1^ Centre for Trials Research, College of Biomedical and Life Sciences, Cardiff University, Cardiff, UK

^2^ Antibiotic Research UK, York, UK

^3^ School of Pharmacy, De Montfort University, Leicester, UK
